# White Matter Integrity in Genetic High-Risk Individuals and First-Episode Schizophrenia Patients: Similarities and Disassociations

**DOI:** 10.1155/2017/3107845

**Published:** 2017-03-19

**Authors:** Yifang Zhou, Jie Liu, Naomi Driesen, Fay Womer, Kaiyuan Chen, Ye Wang, Xiaowei Jiang, Qian Zhou, Chuan Bai, Dahai Wang, Yanqing Tang, Fei Wang

**Affiliations:** ^1^Department of Psychiatry, The First Affiliated Hospital of China Medical University, Shenyang, Liaoning, China; ^2^Department of Geriatrics, The First Affiliated Hospital of China Medical University, Shenyang, Liaoning, China; ^3^Department of Psychiatry, Yale University School of Medicine, New Haven, CT, USA; ^4^Department of Psychiatry, Washington University School of Medicine, St. Louis, MO, USA; ^5^Mental Health Center of Shenyang, Shenyang, Liaoning, China; ^6^Department of Radiology, The First Affiliated Hospital of China Medical University, Shenyang, Liaoning, China

## Abstract

White matter (WM) neuroimaging studies have shown varied findings at different stages of schizophrenia (SZ). Understanding these variations may elucidate distinct markers of genetic vulnerability and conversion to psychosis. To examine the similarities and differences in WM connectivity between those at-risk for and in early stages of SZ, a cross-sectional diffusion tensor imaging study of 48 individuals diagnosed with first-episode SZ (FE-SZ), 37 nonpsychotic individuals at a high genetic risk of SZ (GHR-SZ), and 67 healthy controls (HC) was conducted. Decreased fractional anisotropy (FA) in the corpus callosum (CC), anterior cingulum (AC), and uncinate fasciculus (UF) was observed in both the GHR-SZ and FE-SZ groups, while decreased FAs in the superior longitudinal fasciculus (SLF) and the fornix were only seen in the FE-SZ participants. Additionally, both GHR-SZ and FE-SZ showed worse executive performance than HC. The left SLF III FA was significantly positively correlated with hallucinations, and right SLF II was positively correlated with thought disorder. The presence of shared WM deficits in both FE-SZ and GHR-SZ individuals may reflect the genetic liability to SZ, while the disparate FA changes in the FE-SZ group may represent symptom-generating circuitry that mediates perceptual and cognitive disturbances of SZ and ultimately culminates in the onset of psychotic episodes.

## 1. Introduction

Disconnectivity of different brain regions, which is putatively mediated by white matter (WM) abnormalities, is widely considered to be a key feature of schizophrenia (SZ) [1–3]. Postmortem histological studies support the involvement of WM abnormalities in the pathophysiology of SZ [4, 5]. Decreased expressions of myelin/oligodendrocyte-related genes have been reported in SZ studies [6, 7]. Structural MRI studies have also implicated WM abnormalities in SZ, demonstrating altered WM volumes in multiple lobes of SZ patients [8–10]. Similar WM volumetric changes have been observed in SZ patients experiencing their first psychotic episode (FE-SZ) and in nonpsychotic healthy populations who are at increased genetic high-risk for SZ (GHR-SZ) [11–13]. Recently, diffusion tensor imaging (DTI) has further substantiated WM involvement in SZ. In chronic SZ, DTI studies have detected widespread abnormalities in the WM integrity. The WM bundles that have been most frequently observed to be affected in SZ include the corpus callosum (CC), anterior cingulum (AC), uncinate fasciculus (UF), fornix (FX), and superior longitudinal fasciculus (SLF) [14–18]. Despite common WM abnormalities across the stages of SZ, findings from FE-SZ and GHR-SZ populations have demonstrated patterns of WM abnormalities that are distinct from those observed in chronic SZ [1, 19–21].

Longitudinal studies have observed deviations in cognition, emotion, and behavior in individuals with SZ years before illness onset [22], implicating altered neurodevelopment in individuals with SZ. Consequently, it is likely that disconnectivity among brain regions long predates the symptomatic onset of SZ. Genetic factors appear to contribute significantly to the altered neurodevelopment observed in SZ. Previous observations on first-degree relatives of the SZ patients, including siblings, parents, and monozygotic twin pairs, as well as the offsprings, raised the possibility of identifying endophenotypes of this disease by comparing with HC and SZ [13, 23–26]. Altered WM in SZ's first relatives was reported in frontal lobe [27, 28], hippocampus [28], cingulum angular area [20], anterior limb of the internal capsule [29], areas close to the right middle and superior frontal gyri [20], forceps minor, and SLF [30]. However, these findings were inconclusive because of heterogonous samples, such as the samples with wide age range or chronic illness duration.

Among the first-degree relatives, the offsprings' prevalence is higher than normal people [31], meaning that they are at increased risk for developing SZ in their later life, so they constitute an important high-risk group to probe markers of genetic vulnerability to SZ. However, such markers alone are not sufficient to identify those at the greatest risk for conversion to SZ. The FE-SZ group, compared with chronic SZ, has the advantages that they are in the very early stages of SZ and are free of confounding influence by multiple relapse, long illness duration, and psychotropic medications. FE-SZ individuals represent the transition period from genetic vulnerability to chronic psychotic illness. Comparisons of FE-SZ and GHR-SZ individuals may provide important insight into the brain abnormalities that transform genetic vulnerability to clinical manifestation and disease progression in SZ.

In this study, we examined WM fractional anisotropy (FA) in FE-SZ, GHR-SZ, and healthy control (HC) participants using a voxel-based DTI approach. We opted to apply a voxel-based analysis because it has been shown to be more sensitive to subtle WM differences than other approaches such as tract-based and ROI-based analysis [1]. Furthermore, voxel-based analysis has shown strong correspondence with other analyses in localizing regional WM abnormalities in the studies of chronic SZ [1, 32]. Given the paucity of definitive evidence, it is difficult to hypothesize the specific fiber bundles involved across the different stages of SZ. However, we theorize that shared versus distinct WM abnormalities between GHR-SZ and FE-SZ point to markers of genetic susceptibility and neuropathological processes in SZ, respectively: (1) WM alterations shared between FE-SZ and GHR-SZ, when compared to HC, reflect genetic vulnerability; (2) distinct WM alterations observed only in FE-SZ, when compared to GHR-SZ and HC, represent symptom-generating processes.

## 2. Methods

### 2.1. Subjects

Forty-eight FE-SZ, 37 GHR-SZ, and 67 HC individuals took part in the study. FE-SZ and GHR-SZ participants were recruited at the outpatient clinic of the Department of Psychiatry, First Affiliated Hospital of China Medical University, Shenyang, China. HC participants were recruited from local community via advertisements. The study was approved by the Institutional Review Board of China Medical University. All participants provided written informed consent.

Absence or presence of Axis I and Axis II disorders was confirmed by consensus between two trained psychiatrists using the Structured Clinical Interview for DSM-IV (SCID) [33] or the K-SADS-PL [34] for participants under age 18. Individuals were excluded from the study for any history of substance/alcohol abuse or dependence, neurological disorder, head injury, concomitant major medical disorder that may affect brain microstructure, or contraindications for MRI.

The FE-SZ participants met DSM-IV criteria for schizophrenia, schizophreniform disorder, or schizoaffective disorder without any other Axis I disorders. All FE-SZ participants were in their first psychotic episode with illness duration of less than one year. With regard to medication status, 24 of the FE-SZ participants were antipsychotic-naïve, while the other 24 participants were taking antipsychotic medications. The GHR-SZ participants had one parent with SZ and did not meet criteria for any DSM-IV Axis I disorders. The HC participants were selected to match in age and sex the FE-SZ and GHR-SZ participants. They had no personal or familial history of Axis I psychiatric disorders. Detailed demographic and clinical characteristics are presented in Table 1.

### 2.2. Imaging Acquisition and Processing

All participants were scanned using a GE Signa HDX 3.0T magnetic resonance imaging scanner with a standard 12-channel head coil at The First Affiliated Hospital of China Medical University. Head motion was minimized with foam padding. Diffusion-weighted images were acquired using a spin-echo planar imaging sequence parallel to the anterior-posterior commissure plane with the following parameters: TR = 17000 ms, TE = 85.4 ms, image matrix = 120 × 120, field of view = 240 × 240 mm^2^, 65 contiguous slices of 2 mm without gap, 25 noncollinear directions, and one no diffusion-weighting baseline image.

Image preprocessing was performed using PANDA (http://www.nitrc.org/projects/panda), a fully automated program for processing of brain diffusion images. After motion and eddy current correction were performed, individual FA images of native space were registered to the FA template in MNI (Montreal Neurological Institute) space, followed by resampling the images to a customized spatial resolution 1 × 1 × 1 mm with subsequent warping transformations. Lastly, the FA images were smoothed by a 6 mm Gaussian kernel to reduce noise and misalignment. The resulting images were then used in statistical analyses.

### 2.3. Statistical Analyses

Voxel-based analysis of FA for all WM voxel was performed using analysis of covariance (ANCOVA) in second-level analysis in SPM8 (http://www.fil.ion.ucl.ac.uk/spm) with diagnostic group as an independent factor and age and sex as covariates. Statistical significance was defined by corrected *p* < 0.05 with thresholds at uncorrected *p* < 0.01 and cluster size > 109 voxels as determined by Monte Carlo simulation in AlphaSim [35].

For clusters showing significant differences, FA values were extracted and used in post hoc comparisons. Two-sample *t*-tests of each significant cluster were performed to determine the relative direction of differences between groups (FE-SZ versus HC, GHR-SZ versus HC, and FE-SZ versus GHR-SZ), after controlling for age and sex. Statistical significance was defined by *p* < 0.015 (0.05/3) to achieve Bonferroni correction. The effect of medications was further assessed by comparing nonmedicated versus medicated FE-SZ participants, as well as using the medication as the additional covariate in additional analyses of extracted FAs.

The psychotic symptoms and executive performance were assessed with Brief Psychiatric Rating Scale (BPRS) and Wisconsin card sorting test (WCST). The Pearson's correlation analyses were performed between the FA values showing significant differences among three groups and BPRS (total scores and factor scores) as well as WCST performance.

## 3. Results

**Figure 1:**
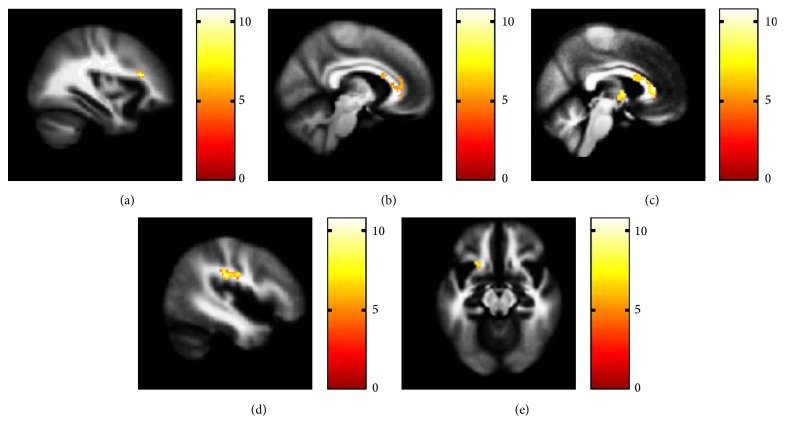
White matter clusters identified in the analysis of covariance (ANCOVA). Images display the white matter bundles yielded from ANCOVA. Fractional anisotropy differs in the (a) right superior longitudinal fasciculus II, *x* = 34 mm, (b) corpus callosum and anterior cingulum, *x* = 8 mm, (c) fornix as well as corpus callosum/anterior cingulum, *x* = 4 mm, (d) left superior longitudinal fasciculus III, *x* = −44 mm, and (e) left uncinate fasciculus, *z* = −14 mm, among the healthy control, genetic high-risk, and first-episode schizophrenia participants.

Significant effect of diagnostic group was observed in 3 clusters: (1) the anterior/posterior genu and body of CC and AC, (2) the right SLF II [SLF subcomponents were defined as four subdivisions in Makris' study [36]], and (3) the left SLF III (MNI coordinates for the point of maximal association, resp.: *x* = 4 mm, *y* = 32 mm, *z* = 8 mm, 458 voxels, and *T* = 4.01; *x* = 34 mm, *y* = 26 mm, *z* = 18 mm, 169 voxels, and *T* = 3.95; *x* = −44 mm, *y* = −24 mm, *z* = 26 mm, 110 voxels, and *T* = 3.75; *p* < 0.05 AlphaSim corrected) (Figure 1). Group differences were also observed in clusters within the FX and UF, although the cluster sizes were smaller than the predetermined threshold (MNI coordinates for the point of maximal association, resp.: *x* = 0 mm, *y* = −2 mm, *z* = 4 mm, 96 voxels, and *T* = 3.67; *x* = −24 mm, *y* = 18 mm, *z* = −14 mm, 47 voxels, and *T* = 3.56; *p* < 0.01, uncorrected).

**Figure 2:**
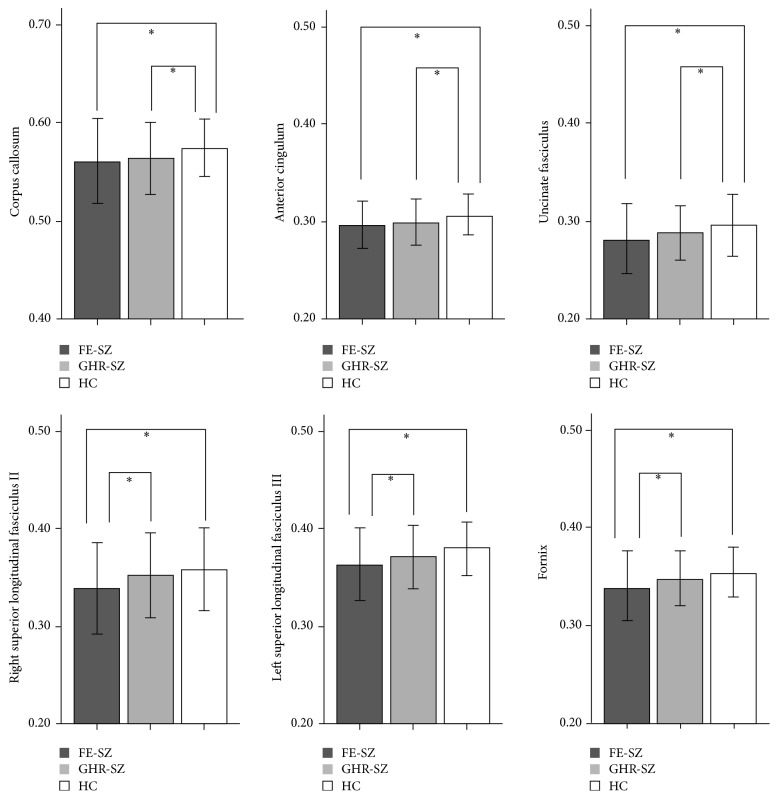
Comparisons of fractional anisotropy (FA) value for white matter clusters of ANCOVA group differences. Shown here are the mean (±standard deviation) FA values extracted from each cluster labeled by the name of white matter bundle and resulting group differences among the first-episode schizophrenia patients (FE-SZ), genetic high-risk individuals (GHR-SZ), and healthy control participants (HC, ^*∗*^*p* < 0.05, corrected by Bonferroni correction).

Post hoc analyses revealed decreased FA in the CC, AC, and UF in both the FE-SZ and GHR-SZ group when compared to the HC group, with no significant difference in FA between FE-SZ and GHR-SZ participants (Figure 2). Significant decrease in FA was found in the right SLF II, left SLF III, and FX in the FE-SZ participants, but not in GHR-SZ participants, when compared to HC (Figure 2).

FAs did not differ significantly between nonmedicated and medicated FE-SZ participants in any WM cluster identified in ANCOVA, indicating no confounding effect from medications. Additionally, when the medication was used as additional covariate, the same results were obtained except CC. Decreased FAs were shown only in GHR-SZ, but not in FE-SZ, when compared to HC.

All the subjects except 5 FE-SZ patients finished the BPRS assessment. 26 FE-SZ, 32 GHR-SZ, and 35 HC finished the WCST (Table 1). We found significant differences among the three groups on BPRS and WCST (except the perseverative errors, *p* = 0.069). Post hoc analyses showed that the BPRS scores were significantly higher in FE-SZ compared to GHR-SZ and HC, but not between GHR-SZ and HC. WCST performance in FE-SZ subjects was worse than GHR-SZ and HC, but no differences existed between GHR-SZ and HC.

Pearson's correlation analyses were performed between the BPRS scores and FA value of left SLF III, right SLF II, and fornix in FE-SZ. The left SLF III FA was significantly positively correlated with hallucinations score of BPRS (*r* = 0.309, *p* = 0.044), and right SLF II was positively correlated with thought disorder subscore (*r* = 0.293, *p* = 0.057). The Pearson's correlation analyses between WCST performance and the FA values of the CC, AC, and UF did not show significant associations.

## 4. Discussion

In this study, we performed a voxel-based DTI analysis to investigate the similarities and differences in WM integrity between the FE-SZ and GHR-SZ participants. We found decreased FA in the CC, AC, and UF in both the FE-SZ and GHR-SZ groups when compared to the HC group, while decreased FA in the right SLF II, left SLF III, and FX was only evident in the FE-SZ group. These findings demonstrate partially overlapped and clearly distinct alterations in WM integrity between FE-SZ and GHR-SZ individuals.

The findings herein agree well with general findings from other DTI studies of disrupted intra- and interhemispheric structural WM connections in SZ. They provide further evidence for the conceptualization of SZ as a disconnectivity syndrome. We observed widespread FA deficits in SZ in WM bundles that connect hetero modal association cortices (HASC), such as the inferior parietal lobule, ventral prefrontal cortex, and superior temporal gyrus. The HASC serve as convergence points for multimodal sensory inputs and organize these inputs to guide responses to environmental stimuli and demands [2]. The dynamics between the HASC ultimately forms an internal abstraction of the external world and mediates adaptation to the external environment, online monitoring of feedback, and interactions between oneself and the environmental context. WM deficits, similar to those found in this study, would likely result in disturbed functional coordination among the HASC, which is a long-held conceptualization of SZ pathophysiology [2, 37]. The subsequent disruption in the orchestration of HASC function could lead to a myriad of cognitive, emotional, and behavioral impairments, as seen in SZ [38, 39].

Decreased FAs in the CC, AC, and UF were observed in both the FE-SZ and GHR-SZ groups, relative to the HC group. Findings of abnormalities in the CC have been fairly consistent across the different stages of SZ [3, 40–43]. In the at-risk populations, a similar voxel-based study found most pronounced changes in the body and splenium of CC in FE-SZ individuals with more modest alterations in at-risk individuals [44]. Other studies have observed abnormalities in the genu of the CC in FE-SZ using region of interest (ROI) and tract-based approaches [41, 45], as well as an association between decreased FA in the CC genu and illness duration in chronic SZ.

Shared WM deficits were also found in the frontotemporal (e.g., the UF) and frontolimbic (e.g., the AC) pathways in the FE-SZ and GHR-SZ groups. Skewed distribution of FA in the left UF has been previously reported in studies of FE-SZ, implicating abnormalities at the center of the UF. Correlation between FA in the UF and negative symptoms, as well as an accelerated decline in UF FA with aging, has been shown in chronic SZ [46]. In our earlier work using a ROI-based approach, we found decreased FA and diminished left-greater-than-right asymmetry in the AC in chronic SZ [14, 15]. The AC connects the anterior cingulate cortex (ACC) with prefrontal limbic regions, the amygdala, and the thalamus [5]. Previous studies have revealed reduced glial cells, cortical thinning, and smaller volume in the ACC of SZ patients [5, 47, 48]. In conjunction with prior evidence, the current findings suggest that disturbances in the CC, AC, and UF indicate genetic susceptibility to SZ and are present before the manifestation of psychosis and persist as the illness progresses.

Distinct from the GHR-SZ group, the FE-SZ group showed additional WM alterations in the right SLF II, left SLF III, and FX, when compared to the HC group. The SLF II and SLF III play overlapping roles in integrating information in the frontoparietotemporal circuitry [36, 49], particularly in auditory perception and speech production. The SLF III originates from rostral inferior parietal lobule and projects to Broca's area, and the SLF II (including the rostral arcuate fasciculus) relays communication between Broca's, Geschwind's, and Wernicke's regions [36]. Hallucination severity has been associated with altered FA in the SLF in SZ, suggesting that the SLF is a neural substrate for auditory hallucination in SZ [46]. The FX carries connections between the hippocampus and hypothalamus. Several lines of evidence have indicated the involvement of hippocampus and FX in SZ pathophysiology. Decreased FA in the FX has been shown in conjunction with bilateral hippocampal volume loss in SZ [50]. Decreased FA in the FX has also been found in chronic SZ along with decreased resting-state functional connectivity between the hippocampus and brain regions subserving episodic memory, including the prefrontal cortex and posterior cingulate cortex [18]. As they were only seen in the symptomatic FE-SZ group and not in the vulnerable GHR-SZ group, we postulate that the findings of FA reductions in the SLF and FX represent symptom-generating WM abnormalities in SZ.

Our findings of shared and distinct abnormalities of WM integrity in FE-SZ and GHR-SZ raise intriguing questions about the role of genetic, neural, and other factors in conversion to psychosis among individuals at-risk for SZ. Are there differential markers for vulnerability to SZ versus definite progression to psychosis? Are certain factors more critical in conversion to psychosis? Our findings appear to suggest that disruptions in the CC, AC, and UF, shared by the FE-SZ and GHR-SZ groups, may represent genetic vulnerability to SZ, but do not result in symptomatic onset of SZ, whereas abnormalities in the right SLF II, left SLF III, and FX, found only in the FE-SZ group, may reflect key neural processes that generate definitive symptoms of SZ. In addition, our findings in the SLF and FX in FE-SZ may represent symptom-generating processes that are present in all psychotic disorders. Studies of these regions in other types of psychosis could provide insight into the level of neural differentiation among psychotic disorders. Our findings also speak to the synergistic effects of susceptibility and illness-generating factors in psychotic conversion of at-risk individuals. Longitudinal studies have shown higher rates of conversion to psychosis when genetic predisposition was coupled with environmental adversities [51]. Conceivably, individuals at-risk for SZ may develop psychosis as a result of dysfunction within key symptom-generating neural circuitry due to further WM disruptions from environmental factors, such as repeated exposure to psychosocial stress, in conjunction with predisposing neural vulnerabilities.

The inclusion of both FE-SZ and GHR-SZ individuals is the major strength of our study. This allows us to dissociate WM abnormalities related to genetic vulnerability and those related to illness itself. Importantly, the SZ participants in our study were in their first episode of illness. Studying those in early stages of illness is substantially advantageous for identifying primary neuropathological processes proximal to initial symptom onset; findings from such samples are not confounded by illness chronicity and recurrence. Half of the FE-SZ participants (24/48) were taking antipsychotic medications at the time of scan. Post hoc analyses indicated no significant effects of medications on our primary WM findings, except CC. How medications influenced CC FA values in SZ needs further investigation in larger sample without taking medications.

While our central findings are consistent with previous literature, there are some differences regarding the laterality of findings in the SLF and UF and the specific subregions of CC that are affected in SZ [3, 40, 43, 45]. We found abnormalities in the genu/body of the CC, whereas other studies have reported alteration in the splenium of the CC [3, 43]. These inconsistencies may arise from the criteria used in identifying at-risk individuals and variations and differences in data processing procedures due to the variety of algorithms and software tools available. Prior studies have varied in using familial predisposition [20, 29] or the presence of prodromal symptoms [44, 52] to determine risk status for SZ. Other potential factors in inconsistencies among studies include age, ethnic make-up of study samples, and acquisition parameters of different MRI scanning systems.

Consistent with the previous studies [53, 54], FE-SZ group demonstrated higher psychotic symptom scores, and positive correlation between the SLF FA values and hallucinations and thought disorder. This suggests that the SLF abnormalities may contribute to the hallucinations and thought disorder associated with schizophrenia. Additionally, we found that both EF-SZ and GHR-SZ showed worse WCST performance, compared to HC, which implicated that the executive performance may be related to the genetic susceptibility to SZ; however, no correlation exists between the WCST performance and FA values of the shared abnormal WM between GHR-SZ and FE-SZ.

## 5. Limitations

Our study is limited by the use of a cross-sectional design. Longitudinal studies are required to track the eventual development of schizophrenia in the high-risk group to confirm that findings are related to risk for the development of schizophrenia rather than protective factors. Second, the HR group in current study included only 37 individuals who are the children of patients with SZ. The sample size may be small considering heterogeneity in this sample, as only a portion of these individuals will later develop SZ. Future studies should distinguish between genetically high-risk relatives without symptoms, and those with existing prodromal symptoms without familial risk. Third, there are some potential problems with the voxel-based DTI analyses that were performed in the study to explore whole brain white matter abnormalities. For example, there is no satisfactory solution to aligning FA images from multiple subjects [55], and the applications of different spatial smoothing extent can give rise to varying results [56]. Further investigation with complementary DTI techniques, such as combining tract-based spatial statistics, would be important.

## 6. Conclusion

In summary, this study lends further support to the critical involvement of WM disruptions in SZ. Importantly, shared and distinct alterations in WM integrity were observed between FE-SZ and GHR-SZ. The shared abnormalities in the CC, AC, and UF may stem from genetic vulnerability to SZ that are present in both FE-SZ and GHR-SZ; whereas the distinct changes in the SLF and FX seen only in the FE-SZ group may point to separate neural mechanisms responsible for psychotic symptoms in SZ, such as hallucinations. Future studies may further elucidate differentiating features between FE-SZ and GHR-SZ, which would improve prediction of psychotic conversion in at-risk individuals. Such differentiation could significantly strengthen the effectiveness of early identification and intervention strategies in SZ, a disorder in which initiation of treatment at the time of psychotic onset is often too late for substantial impact on clinical outcome in affected individuals.

## Figures and Tables

**Table 1:** Demographic and clinical data of participants.

	FE-SZ (*n* = 48)	GHR-SZ (*n* = 37)	HC (*n* = 67)	*p*
Age (year, mean ± SD)	19.6 ± 4.21	19.97 ± 5.36	21.07 ± 4.84	*p* = 0.234
Sex (F/M)	22/26	23/14	35/32	*p* = 0.326
Handedness (R/L/MIX)	44/1/3	33/1/3	64/0/3	<0.609
Medication (yes/no)	24/24	1/36	N/A	
Illness duration (month, mean ± SD)	5.16 ± 9.20	N/A	N/A	
BPRS (mean ± SD)	38.47 ± 13.39^ab^	18.65 ± 1.51	18.01 ± 0.122	<0.000
BPRS (subscore_anxiety/depression)	8.09 ± 4.16^ab^	4.57 ± 1.35	4.00 ± 0.00	<0.000
BPRS (subscore_Lack of energy)	8.51 ± 4.37^ab^	4.00 ± 0.00	4.00 ± 0.00	<0.000
BPRS (subscore_Thought disorder)	10.14 ± 4.56^ab^	4.00 ± 0.00	4.00 ± 0.00	<0.000
BPRS (subscore_activition)	4.70 ± 2.03^ab^	3.00 ± 0.00	3.01 ± 0.121	<0.000
BPRS (subscore_Hostility/suspicion)	7.49 ± 3.75^ab^	3.05 ± 0.33	3.00 ± 0.00	<0.000
Wisconsin_Total Correct	18.58 ± 12.19^a^	22.31 ± 12.89^c^	32.49 ± 11.59	<0.000
Wisconsin_Categories Completed	1.65 ± 1.77^a^	2.63 ± 2.24^c^	4.34 ± 1.96	<0.000
Wisconsin_Total Errors	29.42 ± 12.19^a^	25.69 ± 12.89^c^	15.51 ± 11.59	<0.000
Wisconsin_Perseverative Error	11.96 ± 12.07	11.66 ± 13.13	6.26 ± 7.57	0.069
Wisconsin_Nonperseverative Errors	17.46 ± 7.40^a^	14.03 ± 8.45^c^	9.26 ± 4.59	<0.000

SD: standard deviation.

BPRS: Brief Psychiatric Rating Scale.

FE-SZ: first-episode schizophrenia patients.

GHR-SZ: individuals at genetic high risk for schizophrenia.

HC: healthy controls.

^a^FE-SZ significantly different from HC, *p* < 0.05.  ^b^FE-SZ significantly different from GHR-SZ, *p* < 0.05. ^c^GHR-SZ significantly different from HC, *p* < 0.05.
